# Selective Autophagy as a Potential Therapeutic Target in Age-Associated Pathologies

**DOI:** 10.3390/metabo11090588

**Published:** 2021-08-31

**Authors:** Margarita-Elena Papandreou, Nektarios Tavernarakis

**Affiliations:** 1 Institute of Molecular Biology and Biotechnology, Foundation for Research and Technology-Hellas, 70013 Heraklion, Greece; m.papandreou@imbb.forth.gr; 2 Department of Basic Sciences, Faculty of Medicine, University of Crete, 70013 Heraklion, Greece

**Keywords:** age-related disease, aging, aggrephagy, mitophagy, neurodegeneration, nucleophagy, pexophagy, rapamycin, selective autophagy

## Abstract

Progressive accumulation of damaged cellular constituents contributes to age-related diseases. Autophagy is the main catabolic process, which recycles cellular material in a multitude of tissues and organs. Autophagy is activated upon nutrient deprivation, and oncogenic, heat or oxidative stress-induced stimuli to selectively degrade cell constituents and compartments. Specificity and accuracy of the autophagic process is maintained via the precision of interaction of autophagy receptors or adaptors and substrates by the intricate, stepwise orchestration of specialized integrating stimuli. Polymorphisms in genes regulating selective autophagy have been linked to aging and age-associated disorders. The involvement of autophagy perturbations in aging and disease indicates that pharmacological agents balancing autophagic flux may be beneficial, in these contexts. Here, we introduce the modes and mechanisms of selective autophagy, and survey recent experimental evidence of dysfunctional autophagy triggering severe pathology. We further highlight identified pharmacological targets that hold potential for developing therapeutic interventions to alleviate cellular autophagic cargo burden and associated pathologies.

## 1. Introduction

Cellular garbage disposal is critical for recycling defective cell constituents, such as proteins and organelles, towards the maintenance of cellular homeostasis. One of the main degradative molecule pathways is autophagy, which is a physiological catabolic process shared by all eukaryotes. Derived from the Greek words ‘auto’ meaning self, and ‘phagy’, meaning eating, autophagy, it was initially considered to be a bulk degradation process, while now its highly selective nature is increasingly appreciated. This self-digestive mechanism relieves the cell from proteotoxic, genotoxic, oxidative and nutrient stress [1]. It is accomplished in an intricate stepwise manner, which leads to clearance of damaged cell constituents, in the degradative organelle, the lysosome. Failure to complete this procedure has been implicated in many age-related diseases. Three main types of autophagy have been characterized in detail: macro-autophagy, henceforth referred to as autophagy, which invariably entails the formation of a double membrane vesicle that fuses with the lysosome; micro-autophagy, where there is direct interaction between the autophagic substrate and the lytic organelle, and chaperone-mediated autophagy (CMA), where autophagic substrates are targeted by chaperones and guided to specific receptors on the lysosome, for degradation.

## 2. Main Text

### 2.1. General Autophagy

Autophagy involves three main, consecutive steps: initiation, elongation and autophagosomal/lysosomal fusion. Although basal autophagy occurs at different levels, depending on the tissue, particular stimuli, such as, protein aggregation, DNA damage, reactive oxygen species (ROS), and nutrient deprivation activate or upregulate the autophagic response [2]. Initially, early autophagic structures form, at the pre-autophagosomal site (PAS), where there is nucleation of the initiation membrane, forming the ’moon-shaped’ phagophore. Expansion of the phagophore leads to PI(3)P-rich omegasome formation, which when sealed, forms the double membrane vesicle, the autophagosome. Although the endoplasmic reticulum is the main site for autophagosome formation, ER-mitochondria/plasma membranes contact sites, the plasma membrane itself, the Golgi complex, and recycling endosomes have emerged as autophagosomal biogenesis sites [3,4].

The ULK1 (Unc-51-like kinase 1) and PIKC3-C1 signaling complexes are activated during autophagic induction [5]. Physiologically, phosphorylated ULK1 and ATG13 are inactive and bound to mTORC1 (master cell growth regulator). During amino acid starvation, ULK1 is dephosphorylated and released from mTORC1, which in turn activates ATG13 and FIP200 [6]. Moreover, TFEB is disinhibited upon starvation to upregulate autophagy genes, as well as, lysosomal and lipid catabolism [7]. Next, phagophore expansion involves ATG8 family proteins, which are cleaved by the ATG4 protease at their C-terminus, and then lipidated. Activation of lipidated ATG8s is performed by ATG7 with the aid of ATG5-ATG12. This activity is localized at the phagophore by ATG16L, ultimately leading to phagophore expansion. However, the requirement for ATG8 lipidation for autophagosome assembly has been recently challenged [8].

During autophagosome maturation, the autophagosomal membrane is targeted to the lysosomal membrane by ATGs, the cytoskeleton, mainly microtubule-related kinesins, and the fusion machinery. The fusion machinery comprises SNAREs, both on the autophagosomal, syntaxin 17 (STX17), synaptosomal-associated protein (SNAP29) and lysosomal membrane (VAMP8), with the aid of the homotypic fusion and protein sorting (HOPS) complex, for membrane tethering during fusion [9,10].

### 2.2. Selective Autophagy

Selective autophagy degrades a plethora of autophagic cargo, which is targeted upon specific cellular insults. Defective mitochondria (mitophagy), protein aggregates (aggrephagy) or pathogenic bacteria (xenophagy) are selective autophagy triggers. Atg8 proteins interact and recruit selective autophagic receptors, which contain LIR (LC3-interacting) motifs (W/F/Y-X-X-L/I/V), with upstream negatively charged residues for higher affinity interactions, as well as, post-translational modifications, such as phosphorylation [11]. These receptors are recruited upon induction of selective autophagy, which is, in turn, directed to specific autophagic substrates by other tags, such as K27-linked mono-or K63 poly-ubiquitination events. Autophagic receptors such as p62, NBR1 (a neighbor of BRCA1 gene 1), OPTN1 (optineurin) contain both LIR and UBA binding motifs [12]. ULK1 controls selective autophagy independently of mTOR. Recent evidence suggests that ULK1 interaction with huntingtin is required for activation. Subsequently, huntingtin aids the LC3-p62-autophagic cargo interaction [13,14].

#### 2.2.1. Mitophagy and Aging

Homeostatic mechanisms that respond to mitochondrial damage are less efficient during aging. Mitophagy is a physiological eukaryotic pathway, which involves the degradation of superfluous or damaged mitochondria [15]. When perturbed, normal mitochondrial function is hindered, resulting in the production of excessive ROS [16]. This ultimately leads to cellular dysfunction and tissue damage.

A multitude of mitophagic regulators and receptors have been identified that are cargo content and stress-dependent. Phosphatase and tensin homologue (PTEN)-induced putative kinase 1 (PINK1) and 1-E3 ubiquitin ligase Parkin-mediated mitophagy is the predominant type of autophagic degradation of mitochondria. Under non mitophagy-inducing conditions, PINK is transferred to the inner mitochondrial membrane, where it is cleaved by proteases and subsequently degraded by the proteasome [17,18]. Upon membrane depolarization, PINK1 phosphorylation and Parkin-mediated ubiquitination of outer mitochondrial membrane proteins initiates a series of intricate events, which ultimately leads to autophagic machinery recruitment, for whole mitochondria degradation [17]. In addition, PINK1 indirectly activates dynamin-related protein 1 (DRP1), which in turn promotes fission of defective mitochondria to facilitate mitochondrial autophagic degradation [19].

Although Parkin-dependent mitophagy accounts for a large percentage of mitochondrial recycling, there are other Parkin-independent pathways that involve different ubiquitin ligases. These enzymes generate ubiquitin chains to recruit autophagic adaptors such as optineurin (OPTN), nuclear dot protein 52 (NDP52) and p62, which directly interact with LC3 through their LIRs [14,15]. Moreover, core autophagic components such as the Unc-51-like autophagy activating kinase 1, (ULK1) and the double FYVE-domain containing protein 1 (DFCP1), are also localized close to mitochondria to alleviate mitoaggregation [20]. Outer mitochondrial membrane proteins can act as mitophagy receptors themselves, during cellular homeostasis, differentiation and hypoxia, specifically, NIX (NIP3-like protein X), BNIP3 (BCL2 interacting protein 3) and FUNDC1 (FUN14 domain-containing protein) [21,22,23]. Importantly, NIX and BNIP3 regulate Parkin recruitment, highlighting the interplay between the PINK1-Parkin pathway and mitophagy receptors [24].

**Figure 1:**
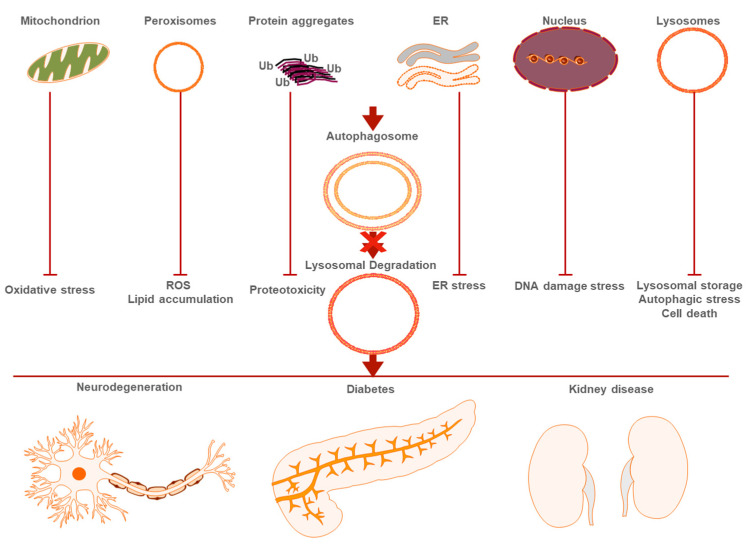
Perturbation of selective autophagy triggers age-related disease. Autophagic degradation of organelles (mitochondria, peroxisomes, ER, nuclei, lysosomes) and protein aggregates leads to loss of cellular homeostasis and subsequent pathological conditions.

Defective mitophagy is evident in a variety of age-related pathologies such as neurodegeneration, metabolic syndromes and myopathies (Figure 1) [25]. In Alzheimer’s disease (AD), PINK1 expression is extremely low, while mitochondrial numbers and oxidative stress increase [26]. Mutations in PINK1/Parkin have been identified in familial Parkinson’s disease, while overexpression of NIX upregulates mitophagy in PINK1/Parkin deficient neurons [27]. Mutations the homologues of these proteins cause decreased lifespan, dopaminergic neuronal death and muscle atrophy, in Drosophila [28]. Defective mitophagy has also been found to induce autoimmune responses, in a cell non autonomous manner. In the absence of PINK1/Parkin, immune cells trigger an immune response by expressing MHC-class I at their plasma membrane [29]. Additionally, retinal ganglion cell axons have been shown to extrude their mitochondria that are then degraded by neighboring astrocytes [30]. In Huntington’s disease, mutant huntingtin appears to perturb mitophagic function [31]. Mitophagy is also essential for cardiac function, and protects against high fat diet, diabetic induced, cardiomyopathy [32]. Recently, PINK1 and parkin have also been associated with mitochondrial genome mutations; mitophagic levels are inversely proportional to mitochondrial DNA mutations especially in neurons [33].

With regard to therapeutic intervention, several pharmacological compounds have been shown to activate mitophagy and alleviate symptoms of age-related diseases, dependent on dysfunctional mitochondria. Similar to aggrephagy, rapamycin activates AMPK, while blocking mTOR, maintaining energetic demands and preventing neurological symptoms, such as neuroinflammation [34,35]. Metformin and pifithrin induce Parkin by inhibiting p53 activity and alleviating diabetic phenotypes [36,37,38]. Resveratrol, mainly found in grape skin, as well as, NAD+ precursors found in natural compounds activate mitophagy and mitochondrial biogenesis through the sirtuin 1 (SIRT1)-PGC-1α axis [39,40]. Urolithin A, an intestinal microbiome-derived metabolite from dietary intake, induces both mitochondrial degradation and biogenesis, and increases health span of model organisms such as *C. elegans* and mice (Table 1) [41].

#### 2.2.2. Aggrephagy and Age-Related Disease

Aggrephagy degrades aggregation-prone proteins via targeted macro-autophagy, in addition to CMA and the proteasomal pathway. These proteins typically form aggresomes near the nucleus, which are surrounded by intermediate filament cytoskeleton, and are further processed to be degraded by autophagy. Protein aggregation usually occurs due to misfolding and can cause, among others, dysregulation of calcium homeostasis, inflammation, neurotoxicity [42]. Normal λy, in unstressed cells, the proteasome is the main degradative pathway for ubiquitin-tagged proteins; however, protein aggregation and overload requires activation of autophagy [1]. Specific neurodegenerative diseases represent prominent examples of dysfunctional aggrephagy. In AD, Parkinson’s disease, amyotrophic lateral sclerosis and polyglutamine diseases, autophagy is perturbed (Figure 1) [43,44]. In the context of these pathologies, defective proteins accumulate in aggresomes, which are identified by their ubiquitination status and the selective autophagic receptor p62, for autophagosomal targeting [45].

Pharmacological compounds, which can upregulate autophagy, with the aim of ameliorating pathology in neurodegenerative diseases, have been identified. In AD, amyloid-beta aggregates in oligomers, autophagy is defective and synapse formation and function are perturbed. The size and solubility of aggregates determines the extent of cellular toxicity effects [46]. AVN-211, Lu AE58054, SB-742457 which are antagonists of the mTOR activator, 5-HT6R, significantly delay memory impairment in AD, with the second reaching Phase III clinical trials, while the latter reaching Phase II, but eventually failing to show the expected efficacy (Table 1) [47,48,49]. Recently, alborixin was identified as an autophagy inducer in both neuronal and glial cells, through the upregulation of several autophagic mediators such as Beclin-1, ATG5 and ATG7, by inhibiting the AKT pathway [50]. Oral administration of a recombinant AAV/Aβ, which alleviated Aβ overload, was also found to activate autophagy [51]. Moreover, rapamycin inhibits mTOR, while resveratrol activates AMPK to induce autophagy. The latter has reached Phase III trial [52]. Lithium, which is used in psychiatric disorder treatments, acts via GSK-3β inhibition to delay cognitive decline [53]. AUTEN-67, an inhibitor of MTMR14, which is antagonistic to autophagosome membrane formation, promotes autophagy, longevity and prevents neuronal cell death, in both in vitro and in vivo AD and Huntington’s disease models [54]. Nicotinamide, which enhances autophagosome/autolysosome acidification, promotes autophagic flux and, hence, ameliorates AD pathology, in mouse disease models [55].

In PD, Beclin 1 gene transfer and overexpression of TFEB have been shown to increase degradation of α-synuclein, and generate promising therapeutic results in mouse models [56,57]. Interestingly, curcumin, prevents oxidative stress and inflammation, while blocking α-synuclein aggregation [58]. The NRF2 activator, dimethyl fumarate (DMF), already used for treatment of multiple sclerosis, was shown to alleviate α-synuclein toxicity [59]. In polyglutamine disorders, mutant forms of several proteins such as ataxin-1 and huntingtin contain expanded polyglutamine repeats, which are cleared out by autophagy [60]. Notably, mutant huntingtin perturbs autophagy, aggravating general protein and microRNA regulator Argonaute 2 clearance defects, causing global dysregulation of miRNA expression [61]. Similarly to AD, AUTEN-67 and rapamycin have been shown to have therapeutic effects in PD animal experimental models [62]. Moreover, trehalose and calpastatin have been shown to alleviate symptoms in rodent models, with calpastatin acting as a calpain inhibitor and activator of autophagy [63,64]. Nevertheless, translational efforts aiming to bring these research results to the clinic have been met with limited success.

#### 2.2.3. Pexophagy and Aging

Recycling of peroxisomes is also regulated by autophagy. These small dynamic single membrane organelles regulate fatty acid oxidation, production of bile acid and other lipids, while also producing reactive oxygen species (ROS), which is neutralized by catalase [65]. Moreover, peroxisomes interact with a multitude of other cellular constituents, such as lipids, the ER and mitochondria [66]. Peroxisome biogenesis can be stimulated by oleic acid, methanol or amines in different yeast species [67,68]. In addition, pexophagy is triggered by feeding yeast with peroxisome-independent carbon sources, while it is inhibited when long fatty acids are abundant [69]. In mammals, ubiquitin acts as a tag for peroxisomal proteins, such as PEX3 and PEX5, that are then recognized by autophagic receptors, including p62 and NBR1, initiating peroxisome lysosomal degradation [70,71]. The PEX2-PEX10-PEX-12 complex functions as an E3 ubiquitin ligase that mediates PEX5 receptor recycling. Depletion of the PEX2-PEX10-PEX-12 complex abrogates starvation-induced pexophagy [72].

Pexophagy and peroxisome biogenesis have recently been implicated with disease. During aging, peroxisomal targeting signal 1 (PTS1) protein import deteriorates and catalase function is diminished. Peroxisomes become more abundant and PEX5 accumulates on their membranes. This causes increased production of ROS, which further blocks peroxisomal protein import and contributes to aging (Figure 1) [73]. Additionally, catalase is gradually excluded from peroxisomes, during cellular senescence [74]. Increased ROS production is a common denominator of perturbations in both peroxisomal recycling and mitophagy, during aging [75]. However, specific induction of intraperoxisomal ROS production causes mitochondrial fragmentation, while catalase inhibition disturbs mitochondrial redox potential. Peroxisome dysfunction may precede mitochondrial dysfunction in certain age-related diseases [76]. Moreover, recent high throughput mass spectrometry analyses showed that 30 peroxisomal proteins decrease with age in *C. elegans* [77].

Post-mortem analysis of Parkinson’s disease patient brains showed a reduction in polyunsaturated fatty acid content, including DHA and arachidonic acid, with concomitant increase in saturated fatty acids, compared to healthy controls. Peroxisomal lipids such as cholesterol are reduced in PD, while its oxidized derivatives correlate with PD pathogenesis and progression. Drugs regulating cholesterol levels appear to alleviate PD symptoms (Table 1). Ethanolamine plasmalogens are undetectable in the blood and brain of PD patients, while supplementation with PPI-1011, which is an ethanolamine plasmalogen precursor, reduces dopamine neuron loss in a PD mouse model [78,79,80].

#### 2.2.4. Nucleophagy and Nuclear Alterations in Aging

Autophagic recycling of the nucleus, or nucleophagy, entails the degradation of multiple compartments of the nucleus, from parts of the nucleolus to the nuclear lamina [81]. Nucleophagy has been described in the context of cancer and neurodegeneration, both of which are also age-associated pathologies.

In yeast, nucleophagy is triggered physiologically, under nutrient stress. Autophagic cargo includes the granular nucleolus, which is targeted by the micro-autophagy receptor Nvj1 and the macro-autophagy receptor Atg39 [82]. Nucleolar size has been established as an accurate aging biomarker in *C. elegans* and mammals. Fibrillarin is a major component of the nucleolus, and a nucleolar marker. Notably, reduction of fibrillarin levels causes nucleolar contraction, and extends lifespan, in worms [83]. By contrast, increasing fibrillarin expression, through inhibition of the fibrillarin translational repressor NCL-1, shortens animal lifespan. Remarkably, long-lived *C. elegans* mutants, such as *daf-2* and *eat-2*, display smaller nucleoli, compared to wild type controls. These long-lived mutant animals are also characterized by higher levels of general and cargo-specific autophagy. Genetic downregulation of autophagy in these mutants abrogates their longevity. Consistently, in Drosophila, pharmacological induction of autophagy by rapamycin reduces nucleolar size in the intestine and fat body [84]. These findings outline an intricate relationship between autophagy, nucleolar size, and longevity.

While nucleophagy has not been directly implicated in aging, severe nuclear envelope and nucleoplasm alterations are observed in old animals. Aged nematodes display nuclear loss or decreased DNA copy number in the intestine [85]. Nucleophagy has also been observed under oncogenic stress, where nuclear LC3 directly interacts with the LIR motif of lamin B. Both LC3 and lamin B are then transported together in the cytoplasm for lysosomal degradation [86]. Oncogenic stress is aggravated during aging. Nucleophagy may serve as a mechanism for damage mitigation in this context. Indeed, lamins accumulate in premature aging syndromes [87]. In addition, damaged DNA that progressively accumulates during aging is recycled through autophagy and the lysosomal enzyme Dnase2a (Figure 1) [88]. Thus, this type of nucleophagy could protect against DNA damage, which directly contributes to aging.

In addition to cancer, nuclear lamina degradation has been implicated in neurodegenerative diseases, such as ataxias, which are characterized by defects in several types of autophagy. In a mouse model of dentatorubral-pallidoluysian atrophy (DRPLA), a polyglutamine repeat-associated ataxia, canonical autophagy is inhibited, while nucleophagy-based Lamin B1 degradation and Golgi membrane-associated excretion is activated [89]. Thus, hijacking of the autophagic machinery causes nuclear defects that lead to cell atrophy and death. This is an example of nuclear lamina recycling deregulation, leading to exacerbated nucleophagy and neurodegeneration.

#### 2.2.5. Other Types of Selective Autophagy in Age-Related Disease

ER-phagy is the selective degradation of parts of the endoplasmic reticulum that contributes to the maintenance of ER homeostasis and recovery after ER stress [90]. Specific receptors of ER-phagy have been identified, including FAM134B, SEC62, RTN3L, CCPG1, ATL3 and TEX264. Lesions in FAM134B impair its autophagic receptor function, facilitating, stress-induced apoptosis and degeneration of sensory neurons, which causes severe sensory and autonomic neuropathy [91,92]. Polymorphisms in this gene have been also associated with vascular dementia [93]. Moreover, ER-phagy was recently shown to degrade mutant NPC1, a protein involved in intracellular lipid trafficking, which has been implicated in Niemann-Pick type C, a fatal neurodegenerative disease, [94]. In addition, ATL3 protects sensory neurons by regulating ER membrane-forming proteins, in the absence of which, axonal degeneration ensues [95]. Furthermore, RTN3 deficiency further aggravates amyloid-β deposition, in an AD mouse model [96]. However, the mechanistic association of ERphagy with the aging process itself is not well-understood.

Lysosomal disintegration occurs during aging. Apart from blocking the breakdown of cellular compartments, defects in lysophagy, the recycling of lysosomes; can activate diverse cell death pathways such as apoptosis, necroptosis, pyroptosis and ferroptosis [97]. Notably, in AD lysosomal pH is elevated, impairing the function of the organelle [98]. Moreover, lysosomal cathepsin D activity is required for efficient clearance of α-synuclein in Parkinsons’ disease [99]. Several potential pharmacological compounds which modulate lysophagy have been shown to ameliorate metabolic disorders, such as, diabetes and associated kidney disease (Table 1) [100,101,102,103,104,105,106].

**Table 1:** Association of selective autophagy inducers with disease therapy or aging.

Autophagy Inducer	Type of Autophagy	Disease/Aging	Organism	Reference
Pifithrin	Mitophagy	Diabetes, PD	Mouse	[35]
Metformin	Mitophagy	Diabetes, PD	Mouse, Human	[36]
Urolithin A	Mitophagy	Aging	Mouse	[40]
SB-742457	Aggrephagy	AD	Human	[46]
Lu AE58-54	Aggrephagy	AD	Human	[47]
AVN-211	Aggrephagy	AD	Mouse	[48]
rAAV	Aggrephagy	AD	Mouse, Rat	[50]
Resveratrol	Mitophagy, Aggrephagy Lysophagy	AD, Diabetic kidney disease	Human	[38,51,99]
Rapamycin	Mitophagy, Aggrephagy	AD	Mouse	[34,51]
Lithium	Aggrephagy	AD	Human	[52]
Nicotinamide	Aggrephagy	AD	Human	[54]
DMF	Aggrephagy	PD	Mouse	[58]
Curcumin	Aggrephagy, Lysophagy	PD, Diabetes	Mouse, Rat	[57,104]
Beclin-1 and TFEB overexpression	Aggrephagy	PD	Mouse	[55,56]
AUTEN-67	Aggrephagy	AD, PD, Huntington’s disease	Mouse	[53,61]
Trehalose	Aggrephagy	PD	Mouse	[62]
Calpastatin	Aggrephagy	PD	Mouse	[63]
PPI-1011	Pexophagy	PD	Mouse	[78]
Catalase	Lysophagy	Diabetic kidney disease	Human	[100]
Tubastatin A	Lysophagy	Diabetic kidney disease	Rat	[101]
Torin 1	Lysophagy	Diabetes	Mouse	[102]
Tocopherol	Lysophagy	Diabetes	Rat	[103]

## 3. Conclusions

Extensive research has revealed the direct association of selective autophagy defects and age-related disease. Initially thought to be non-selective, autophagy was considered to be a highly promising therapeutic target. Diseases associated with physiological aging such as neurodegeneration and metabolic disorders are the outcome of genetic inhibition of selective autophagy, which also declines physiologically during aging. Experimental evidence is increasingly showing the significance of autophagic degradation in maintaining organismal homeostasis, particularly in highly specialized tissues such as the nervous system. The intricacy and crosstalk of these selective autophagic pathways raises the challenge of combinatorial drug treatment.

Selective autophagic induction by genetic intervention or chemical compound administration is currently being investigated in multiple diseases as potential therapeutic approach, although no drug has reached the clinic yet. Indeed, clinical studies concerning druggable autophagy targets, remains limited. This highlights the complexity and intricacies of selective autophagic pathways, which in humans, cannot be easily targeted due to context-dependence and extensive crosstalk with other functional networks. Thus, initial optimism has subsided, with research now focusing on specific compounds that could target specific aspects of selective autophagy. An important objective of the collective efforts of the research community and pharmaceutical companies is to achieve targeting selective autophagy mediators, while not affecting other cellular processes. This would be an imperative step, minimizing adverse consequences to organismal physiology, towards clinical trials in human patients.

## Data Availability

Not applicable.
